# Isoflurane promotes phagocytosis of apoptotic neutrophils through AMPK-mediated ADAM17/Mer signaling

**DOI:** 10.1371/journal.pone.0180213

**Published:** 2017-07-03

**Authors:** Xueke Du, Chunling Jiang, Yang Lv, Randal O. Dull, You-Yang Zhao, David E. Schwartz, Guochang Hu

**Affiliations:** 1 Department of Anesthesiology, University of Illinois College of Medicine, Chicago, Illinois, United States of America; 2 Affiliated Tumor Hospital of Guangxi Medical University, Nanning, Guangxi, China; 3 Department of Pharmacology, University of Illinois College of Medicine, Chicago, Illinois, United States of America; 4 Center for Lung and Vascular Biology, University of Illinois College of Medicine, Chicago, Illinois, United States of America; 5 The Affiliated Hospital of Xuzhou Medical University, Xuzhou, Jiangsu, China; Centre National de la Recherche Scientifique, FRANCE

## Abstract

A patient’s recovery from lung inflammatory injury or development of multi-system organ failure is determined by the host’s ability to resolve inflammation and repair tissue damage, both of which require the clearance of apoptotic neutrophils by macrophages (efferocytosis). Here, we investigated the effects of isoflurane on macrophage efferocytosis and resolution of lung inflammatory injury. Treatment of murine bone marrow-derived macrophages (BMDMs) or alveolar macrophages with isoflurane dramatically enhanced phagocytosis of apoptotic neutrophils. Isoflurane significantly increased the surface expression of the receptor tyrosine kinase Mer in macrophages, but markedly decreased the levels of a soluble form of Mer protein in the medium. Isoflurane treatment also caused a decrease in a disintegrin and metalloproteinase 17 (ADAM17) on the cell surface and a concomitant increase in its cytoplasmic fraction. These responses induced by isoflurane were completely reversed by a pharmacological inhibitor or genetic deletion of AMP-activated protein kinase (AMPK). In a mouse model of lipopolysaccharide-induced lung injury, isoflurane accelerated the recovery of lung inflammation and injury that was coupled with an increase in the number of alveolar macrophages containing apoptotic bodies. In alveolar macrophage-depleted mice, administration of isoflurane-pretreated BMDMs facilitated resolution of lung inflammation following lipopolysaccharide challenge. Thus, isoflurane promoted resolution of lipopolysaccharide-induced lung inflammatory injury via enhancement of macrophage efferocytosis. Increased macrophage efferocytosis following isoflurane treatment correlates with upregulation of Mer surface expression through AMPK-mediated blockade of ADAM17 trafficking to the cell membrane.

## Introduction

Acute lung inflammation is an essential defense response to pathogenic or noninfectious insults and is characterized by exudation of intravascular fluid and increased polymorphonuclear neutrophil (PMN) infiltration into the lung interstitial and alveolar space. Recruited PMNs at sites of infection kill and ultimately clear invading microorganisms, which limits the inciting injury or infection [1, 2]. However, uncontrolled and massive inflammatory response can result in lung injury that severely impairs gas exchange. Unresolved lung inflammation also contributes directly to the development of acute respiratory distress syndrome (ARDS) [1, 3]. Thus, successful resolution of lung inflammation, characterized by clearance of inflammatory cells and restoration of alveolar function, is critical for the return of respiratory homeostasis and recovery from acute lung injury [4, 5].

Efferocytosis, or phagocytosis of apoptotic cells by macrophages and other phagocytic cells, is a multi-step process involving sensing, recognition, engulfment, and digestion of dying cells by phagocytes [1, 3]. PMNs in the tissues undergo apoptosis during the resolution of inflammation and then are removed by macrophages to prevent bystander tissue injury from the leakage of noxious contents of dying cells and to limit proinflammatory responses from the ingesting phagocyte [6, 7]. At the early stage of efferocytosis, both exposure of phosphatidylserine (PS) to the exofacial leaflet of the phospholipid membrane and the release of chemokines [5, 8], or “Find Me” signals, by apoptotic cells recruit macrophages to sites where cell death may be occurring. In response, the macrophage increases the expression of efferocytosis receptors and bridge molecules required for the recognition and binding of apoptotic cells. Finally, apoptotic cells are phagocytosed and digested by macrophages during “Eat Me” step. In contrast to phagocytosis of pathogens, which causes proinflammatory response [9, 10], efferocytosis activates anti-inflammatory and pro-resolving signaling pathways that are crucial for resolution of inflammation [11].

The protective effects of volatile anesthetics, including isoflurane, on lung inflammatory injury have been well studied *in vitro* and *in vivo* [12–17]. Isoflurane has been reported to inhibit the proinflammatory cytokine release from alveolar macrophages and monocytes following lipopolysaccharide (LPS) challenge [13, 16–18]; however, the effect(s) of isoflurane on the resolution of lung inflammation remains unknown. In the present study, we identified that isoflurane promoted macrophage efferocytosis and subsequent resolution of lung inflammation in a model of sepsis with endotoxin and that these effects are closely associated with upregulation of receptor tyrosine kinase Mer surface expression in macrophages.

## Materials and methods

### Mice

C57BL/6J mice were obtained from The Jackson Laboratory (Bar Harbor, ME, USA) and inbred in microisolator cages under specific pathogen-free conditions, fed with autoclaved food. All animal procedures were approved by the Institutional Animal Care and Use Committee. Mice of both sexes were used to rule out gender-specific effects. Mice were monitored at least once daily for up to 9 days. General observation used to assess animal health and well-being include change in body weight, external physical appearance, clinical signs, changes in unprovoked behavior, and responses to external stimuli. The mice were sacrificed to obtain lung tissue samples under anesthesia with Ketamine/Xylazine.

### Isolation and culture of BMDMs

BMDMs were isolated from mice as described previously [19]. Briefly, bone marrow was flushed from femurs and tibias with 2 to 5 ml of PBS (without Ca^2+^ and Mg^2+^) using a 25-gauge needle. Bone marrow cells were collected, centrifuged for 10 min at 500 × g at room temperature, and then resuspended in macrophage complete medium (DMEM/F12 with 10% FBS, 20% L-929 cell conditioned medium, 10 mM L-glutamine, 100 IU/ml penicillin, and 100 mg/ml streptomycin). Collected cells were finally plated into a sterile plastic petri dish in macrophage complete medium and incubated at 37°C and 5% CO_2_. Cell culture medium was subsequently replaced on day 3 with fresh complete medium. After 1 week of culture, the adherent cells in culture were >95% pure macrophages, as assessed by the expression of cell-surface markers HLA-DR, CD11b, and CD206, and these BMDMs were used for all subsequent experiments.

### Isolation and culture of mouse alveolar macrophages

Alveolar macrophages were isolated and cultured as described previously [20]. Briefly, after mice were anesthetized with 75 mg/kg ketamine and 3 mg/kg xylazine (i.p.), the lungs were excised en bloc with the trachea, washed in HBSS, and lavaged more than 10 times by slowly instilling and withdrawing 1 ml of warm (37°C) Ca^2+^/Mg^2+^-free HBSS (pH 7.4) containing EDTA (0.6 mM). BAL fluid collected from each group was centrifuged at 400 g for 10 min at 4°C. The cells were seeded in sterilized polystyrene Petri dishes for 2 h at 37°C. The cells adhering to dishes were collected and then replated for further experimental use. The purity of alveolar macrophages was >95% as verified by the surface expression of antigens F4/80 and CD11b.

### Isolation of PMNs and induction of apoptosis

Murine PMNs were isolated from peripheral blood by the Ficoll-Histopaque approach as described previously [21]. PMN apoptosis was induced by exposure of cells to UV irradiation (254 nm, UVS-26, 6W bulb 0.02J/s/cm^2^) for 15 min followed by incubation for 3 h at 37°C in CO_2_ incubator (5% v/v). To confirm PMN apoptosis, cells (10^6^) were stained with annexin V—FITC (BD Biosciences, San Jose, CA, USA) and propidium iodide and then analyzed by flow cytometry within 30 min of staining without fixation. PMNs were approximately 91% apoptotic.

### Treatment of macrophages with isoflurane

The 6-cm dishes or 24-well cell culture plates containing BMDM or alveolar macrophage monolayers in phosphate buffer saline (PBS, Gibco, Langley, OK, USA) were placed in a layer of water within the airtight exposure chamber in a water bath heated to 37°C [22]. Carrier gas (air) was passed through a calibrated vaporizer (Dräger, Lübeck, Germany) providing 0.5~2.0 minimum alveolar concentration (MAC) of isoflurane (1.0 MAC = 1.3 Vol%) [23] at a flow rate of 5 L/min into the airtight chamber.

### Macrophage efferocytosis *in vitro*

Apoptotic PMNs were labeled by incubation of cells with CellTracker^™^ Red (Invitrogen, Carlsbad, CA, USA) at 37°C for 20 min. Mouse BMDMs or alveolar macrophages were labeled with CellTracker^™^ Green (Invitrogen, Carlsbad, CA, USA) at 37°C for 30 min. Macrophages were then incubated with apoptotic PMNs (1:10 ratio) for 2 h. Free and non-ingested apoptotic PMNs were removed by washing three times with ice-cold PBS. The macrophages engulfing apoptotic PMNs were analyzed using fluorescence microscopy. The phagocytosis index is expressed as the percentage of macrophages containing at least one ingested PMN.

### *In vivo* efferocytosis by alveolar macrophages

The lungs were aspirated 3 times with 1 ml of sterile PBS per mouse to collect bronchoalveolar lavage (BAL) fluid. The BAL fluid was pooled and centrifuged, and cell pellets were suspended in PBS. Cell suspensions were cytospun onto slides with a cytocentrifuge (Shandon, Southern Sewickley, PA, USA). Slides were stained with Diff-Quick dye (Dade Behring, Newark, DE, USA) and examined at magnifications of 20× and 40× by light microscopy. At least 300 macrophages were counted in each sample. Phagocytosis was expressed as the percentage of alveolar macrophages containing apoptotic bodies [24].

To assess the alveolar macrophages’ ability to remove apoptotic PMNs *in vivo*, apoptotic mouse PMNs (1.0 × 10^7^ in 100 μl) were labeled with pHrodo^™^ Red (SE) (Invitrogen, Carlsbad, CA, USA) and intratracheally instilled into mice [25]. The fluorescence of pHrodo^™^ Red dramatically increases in phagosomes of macrophages as pH decreases from neutral to acidic. Attached apoptotic PMNs were excluded because pHrodo-SE was nonfluorescent at neutral pH. After 3 hours of PMN instillation, BAL was performed. Cells in BAL fluid were washed, resuspended and analyzed by flow cytometry.

### AMPK knockdown in macrophages

Murine BMDMs at 50–70% confluence were transfected with 25 nM AMP-activated protein kinase (AMPK) small interfering RNA (siRNA) or non-targeting control siRNA (Dharmacon, Lafayette, CO, USA) according to the manufacturer’s protocol. All relevant experiments were performed 48 h posttransfection.

### Flow cytometry

Cells were stained with annexin V—FITC (BD Biosciences, San Jose, CA, USA) and propidium iodide. Unspecific binding was blocked by Fc receptors with 1.5 mg/ml mouse IgG. PMN apoptosis was analyzed by flow cytometry (Becton Dickinson LSR I, San Jose, CA, USA). For determination of *in vivo* efferocytosis, BAL was performed at the end of experiments. Macrophages containing apoptotic mouse PMNs labeled with pHrodo^™^ Red in BAL fluid were assessed by flow cytometry.

For measurement of surface expression of Mer, BMDMs were washed with cold isotonic staining buffer (PBS with 0.5% BSA without Ca^2+^ and Mg^2+^) and harvested by centrifugation at 300 x g for 5 min. The pellets were resuspended in cold staining buffer at a final cell concentration of 1 x 10^6^/100 μl. BMDMs were pre-incubated with anti-mouse CD16/CD32 monoclonal antibody (eBioscience) to block non-specific Fc-mediated interactions followed by labeled with mouse Mer PE-conjugated antibody or rat IgG2A PE-conjugated antibody (R&D system) for 30 min. All samples were acquired on an LSRFortessa flow cytometer (BD Bioscience) and analyzed using FlowJo software.

### Western blotting analysis

Cells were lysed by radioimmunoprecipitation assay (RIPA) buffer supplemented with 1 mM PMSF, 1 mM Na_4_VO_3_, protease and phosphatase inhibitor cocktails. Membrane extracts were prepared using Membrane Protein Extraction Kit (Thermo Scientific, Rockford, IL, USA) according to the manufacturer’s instructions. The cell lysates were sonicated for 10 s and centrifuged at 10,000 × g for 10 min at 4°C. Protein concentration was measured using a Bio-Rad protein assay kit (Bio-Rad Laboratories, Inc, Hercules, CA, USA). Cell extracts (up to 30 μg protein) were separated on PAGE (10–12%) electrophoresis and blotted on nitrocellulose membranes (Invitrogen, Carlsbad, CA, USA). The membranes were probed with primary antibodies overnight at 4°C and then incubated with horseradish peroxidase (HRP)-conjugated secondary antibodies (1:2000~4000) at room temperature for 1 h. The protein bands were detected with Odyssey Fc Imager (LI-COR Biosciences, Lincoln, NE, USA). Relative band densities of the various proteins were measured from scanned films using ImageJ Software (National Institutes of Health) [19, 20].

### Depletion of alveolar macrophages in mice

Alveolar macrophages were depleted via intratracheal instillation of clodronate-containing liposomes (Encapsula NanoSciences LLC, Nashville, TN, USA). Mice were anesthetized with ketamine/xylazine (i.p., 75/3 mg/kg, Sigma, MO) and suspended a 45° angle by the incisors from a rubber band attached to a Plexiglas support. The vocal chords were visualized using a metal “laryngoscope” by lifting the lower jaw of the mouse and keeping the tongue displaced. Fiber-optic light source is adjusted just below vocal cords to provide the best view of trachea. A 2-cm-long PE-60 catheter attached to the hub of a needle was inserted ~3 mm into the trachea. A bolus of liposome-encapsulated clodronate (1.0 mg liposome-encapsulated clodronate in 100 μl total volume, diluted in sterile saline) was injected into the lungs during an inspiratory phase of the breathing cycle. After 15–20 min the animals were usually sufficiently recovered to get up and move around the chamber. Lavageable alveolar macrophage count was reduced by 95% at 2 d following administration of clodronate liposome.

### Murine model of LPS-induced acute lung injury

*Experimental protocol 1*: Mice were anesthetized with ketamine (75 mg/kg) and xylazine (10 mg/kg) and challenged with LPS (*E*. *Coli*. O55:B5, L2880, 3.5 mg/kg in 100 μl, i.t.) (Sigma-Aldrich, St. Louis, MO, USA) or PBS (100 μl, i.t.). On day 3, mice were exposed to 1.0 MAC isoflurane or air in an airtight box for 1 h. On day 5, 7 or 9 following LPS challenge, mice were sacrificed and lung injury was assessed by analysis of BAL fluid, wet/dry lung weight ratio measurement, and biochemical/immunological analysis of lung tissues.

*Experimental protocol 2*: Mice were anesthetized with ketamine (75 mg/kg) and xylazine (10 mg/kg) and depleted of alveolar macrophages by administering clodronate liposome. At 2 d post alveolar macrophage depletion, mice were anesthetized and challenged with LPS (3.5 mg/kg in 100 μl PBS, i.t.) or PBS (100 μl, i.t.). At day 3 after LPS challenge, BMDMs (2 × 10^6^ cells, 40 μl total volumes each) pretreated with 1.0 MAC isoflurane or air for 1.0 h were given to alveolar macrophage-depleted mice via intratracheal instillation. BMDMs were isolated and cultured as described above. On day 5, 7 or 9 following LPS challenge, mice were sacrificed and lung injury was assessed [26].

### Lung tissue myeloperoxidase activity

Myeloperoxidase (MPO) activity in the lung as a marker of PMN infiltration was measured using MPO assay kit (Abcam, Cambridge, MA, USA) according to manufacturer’s instruction [27]. Briefly, lung tissue (~50 μg) was homogenized in 1 ml of cold sample buffer and centrifuged at 16,000 g for 30 min at 4°C. MPO activity was assessed in 100 μl of supernatants in duplicate using development reagent at 450 nm and expressed as a change in absorbance/mg protein.

### Quantification of PMN infiltration in the lung

At the end of the experiments, the absolute number of PMNs in BAL fluid was counted as described previously [27]. In brief, the collected BAL fluid was centrifuged and cell pellets were suspended in PBS. Cell suspension (300 μl) was cytocentrifuged onto a glass slide with a cytocentrifuge (Shandon, Southern Sewickley, PA, USA). Slides were stained with Diff-Quick dye (Dade Behring, Newark, DE, USA) and analyzed by light microscopy (magnification, 20× and 40×). The percentage of PMNs was calculated after counting at least 300 cells in five or more randomly selected fields in each slide.

### Assessment of lung vascular permeability and edema formation

The total protein content in BAL was measured using a Bio-Rad protein assay kit (Bio-Rad Laboratories, Inc, Hercules, CA, USA) to evaluate permeability of the alveolar-capillary barriers [27]. Wet-to-dry lung weight ratio was used as an index of lung edema formation. At the end of experiments, lungs were weighed, dried, and reweighed.

### Lung histology and lung injury scoring

The lungs were fixed by intratracheal instillation of 4% paraformaldehyde (Sigma-Aldrich, St. Louis, MO, USA) in PBS at a constant pressure of 25 cm H_2_O pressure and immersed in fixative overnight. The fixed lungs were washed and then dehydrated in 70% ethanol before paraffin embedding. Lung tissue sections were cut at 4.0-μm thickness for staining with haematoxylin and eosin.

The severity of lung tissue injury was evaluated by two independent, blinded investigators based on the histological semi-quantitative scoring system as described previously [28]. Within each field (magnification, 20×), points were assigned on a scale from 1–5 according to the following criteria: 1, normal; 2, focal (<50% lung section) interstitial congestion and inflammatory cell infiltration; 3, diffuse (>50% lung section) interstitial congestion and inflammatory cell infiltration; 4, focal (<50% lung section) consolidation and inflammatory cell infiltration; and 5, diffuse (>50% lung section) consolidation and inflammatory cell infiltration. The mean injury score from the two investigators was used for comparison between groups.

### Cytokine assays

The levels of proinflammatory cytokines TNF-α and IL-6 as well as anti-inflammatory cytokines active transforming growth factor (TGF)-β1 and IL-10 in BAL fluid were assessed with commercial ELISA kits (Biolegend, San Diego, CA, USA). Each value represents the mean of triplicate determinations.

### Statistical analysis

One-way ANOVA and Student Newman—Keuls test for post hoc comparisons were used to detect differences between control and experimental groups. Parameter changes between different groups over time were evaluated by a two-way ANOVA with repeated measures. Data are expressed as mean ± SEM. Statistical significance was defined as a *P* value of less than 0.05.

## Results

### Isoflurane enhances phagocytosis of apoptotic PMNs by mouse BMDMs and alveolar macrophages

**Fig 1:**
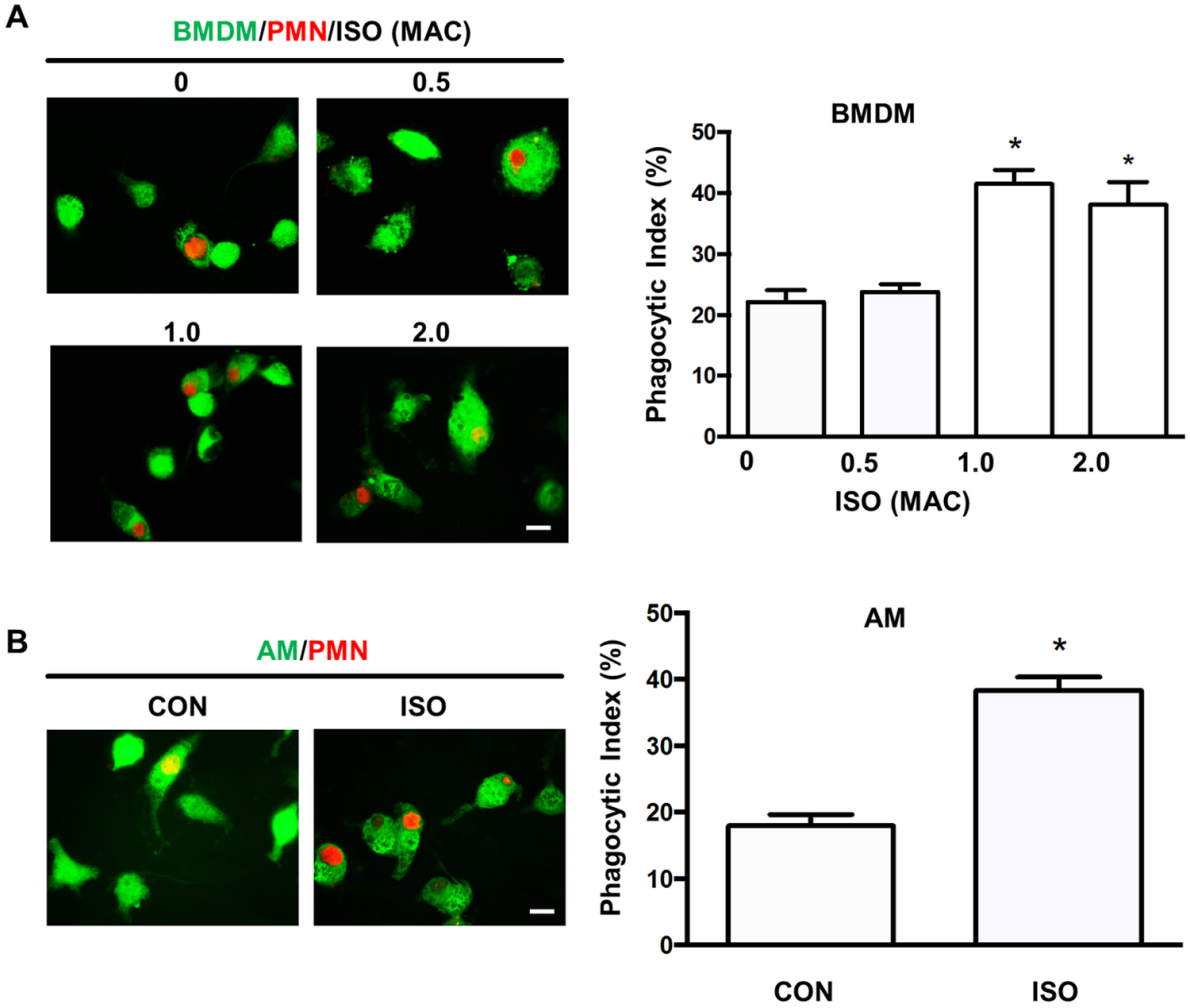
Isoflurane enhances macrophage phagocytosis of apoptotic PMNs. BMDMs, alveolar macrophages and neutrophils (PMNs) were isolated from mice. Macrophages were labelled with CellTracker^™^ Green and incubated with ultraviolet-induced, CellTracker^™^ Red-labelled apoptotic PMNs (A, B) at 1:10 ratio for 2 h. The cells were then mounted on a slide and analyzed by fluorescence microscopy. (**A**) Effects of isoflurane on the phagocytosis of apoptotic PMNs by BMDMs. BMDMs were treated with isoflurane for 1 h at different concentrations (0, 0.5, 1.0 or 2.0 MAC). Left, representative fluorescent images showing macrophages engulfing apoptotic PMNs. Scale bars, 10 μm; right, phagocytic index based on the fluorescent images. (**B**) Effects of isoflurane on the phagocytosis of apoptotic PMNs in alveolar macrophages (AMs). AMs were treated with 1.0 MAC isoflurane for 1 h. Left, representative fluorescent images showing macrophages engulfing apoptotic PMNs Scale bars, 10 μm; right, phagocytic index based on the fluorescent images. Data are mean±SEM of three independent experiments. *P < 0.05 vs. control groups (no isoflurane).

The phagocytic function of macrophages plays a pivotal role in eliminating apoptotic cells and resolving inflammation [2, 29]. Here, we explored the potential effect of the volatile anesthetic isoflurane at clinically relevant concentrations on phagocytosis of apoptotic PMNs by macrophages. We observed that 1.0 MAC and 2.0 MAC isoflurane equally increased efferocytosis in mouse BMDMs in comparison with vehicle (air) (Fig 1A). Consistently, treatment of alveolar macrophages with 1.0 MAC isoflurane also increased phagocytosis of apoptotic PMNs (Fig 1B). Isoflurane at the concentration of 0.5 MAC had no effect on macrophage efferocytosis in both BMDMs and alveolar macrophages (Fig 1A and 1B). These results demonstrate the ability of isoflurane to stimulate macrophage phagocytosis of apoptotic PMNs.

### Isoflurane upregulates the cell surface and total cellular expression of Mer protein in macrophages

**Fig 2:**
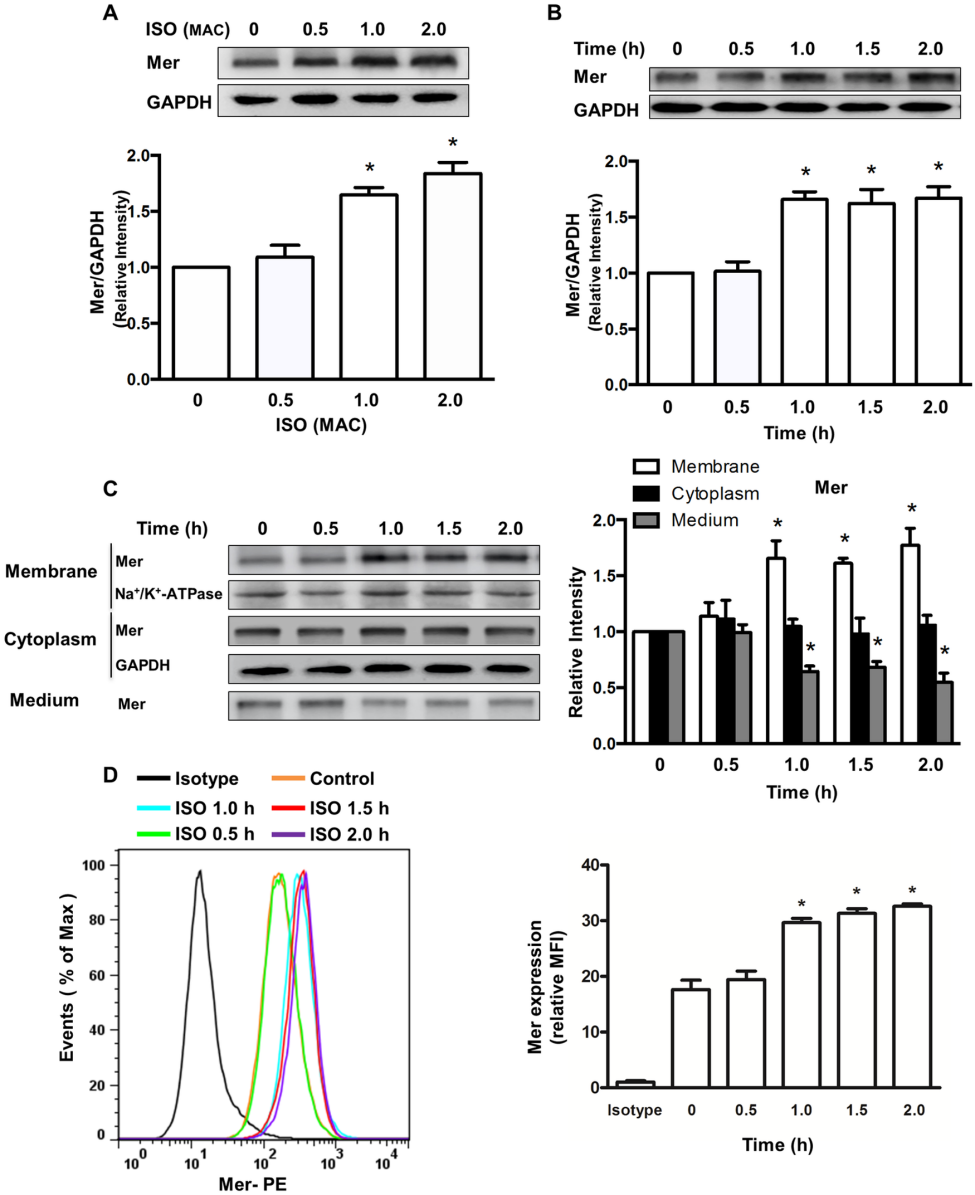
Isoflurane increases the total and surface expression of Mer protein. Mer protein expression was detected by Western blot analysis. (**A**) Effects of isoflurane increased the total expression of Mer protein in a concentration-dependent manner. BMDMs were treated with isoflurane for 1 h at different concentrations. Upper, representative blots showing protein expression; bottom, quantification of Mer protein expression by densitometry. Data are mean±SEM of 3 independent experiments. *P < 0.05 vs. control groups (no isoflurane). (**B**) Time course of total Mer expression induced by isoflurane. BMDMs were treated with 1.0 MAC isoflurane (ISO) for the indicated time points at 37°C. Upper, representative blots showing protein expression; bottom, quantification of Mer protein expression by densitometry. Data are mean±SEM of 3 independent experiments. *P < 0.05 vs. control groups (no isoflurane). (**C**) Effects of isoflurane on the surface and cytosolic expression of Mer as well as a soluble Mer protein in medium. Left, representative blots showing protein expression; right, quantification of Mer protein expression by densitometry. Data are mean±SEM of 3 independent experiments. *P < 0.05 vs. corresponding control groups (no isoflurane). (**D**) Effects of isoflurane on cell surface of Mer expression. Mer expression on the surface was measured by flow cytometry. Left, representative flow cytometry histograms; right, quantitative data showing changes in relative mean fluorescence intensity (MFI) of Mer. Data are presented as mean ± SEM, n = 3. *P < 0.05 versus control group (no isoflurane).

Macrophages express a variety of receptors on the cell membrane that recognize and engage apoptotic cells via direct or indirect binding to ligands on apoptotic cell surfaces. Mer (also known as MerTK), a member of the TAM (Tyro3/Axl/Mer) receptor tyrosine kinase family, is critical for many aspects of immune function by triggering phagocytosis of apoptotic cells by macrophages [30]. In the present study, we examined whether the expression of Mer receptor in macrophages is modulated by isoflurane. Compared to vehicle, both 1.0 and 2.0 MAC isoflurane significantly increased the total expression of Mer protein (Fig 2A). Isoflurane had no effect on Mer expression at the concentration of 0.5 MAC (Fig 2A). The total expression of Mer was found to increase in a time-dependent manner following 1.0 MAC isoflurane treatment (Fig 2B). Since surface expression of Mer serves as a key role in macrophage efferocytosis [30], we further analyzed the effect of isoflurane on Mer expression on the cell surface. Following treatment of BMDMs with 1.0 MAC isoflurane, Mer expression in membrane fractions notably increased at 1 h and maintained a stable level, at least until 2 h (Fig 2C), while lower levels of a soluble form of Mer protein in medium were detected (Fig 2C). However, Mer expression in cytosolic fractions was not altered (Fig 2C). Increased levels of cell surface Mer expression following isoflurane treatment was further verified using flow cytometry (Fig 2D). These data raise the possibility that isoflurane may upregulate Mer surface expression in macrophages via inhibition of Mer cleavage.

### Isoflurane blocks ADAM17 trafficking to cell surface in macrophages

**Fig 3:**
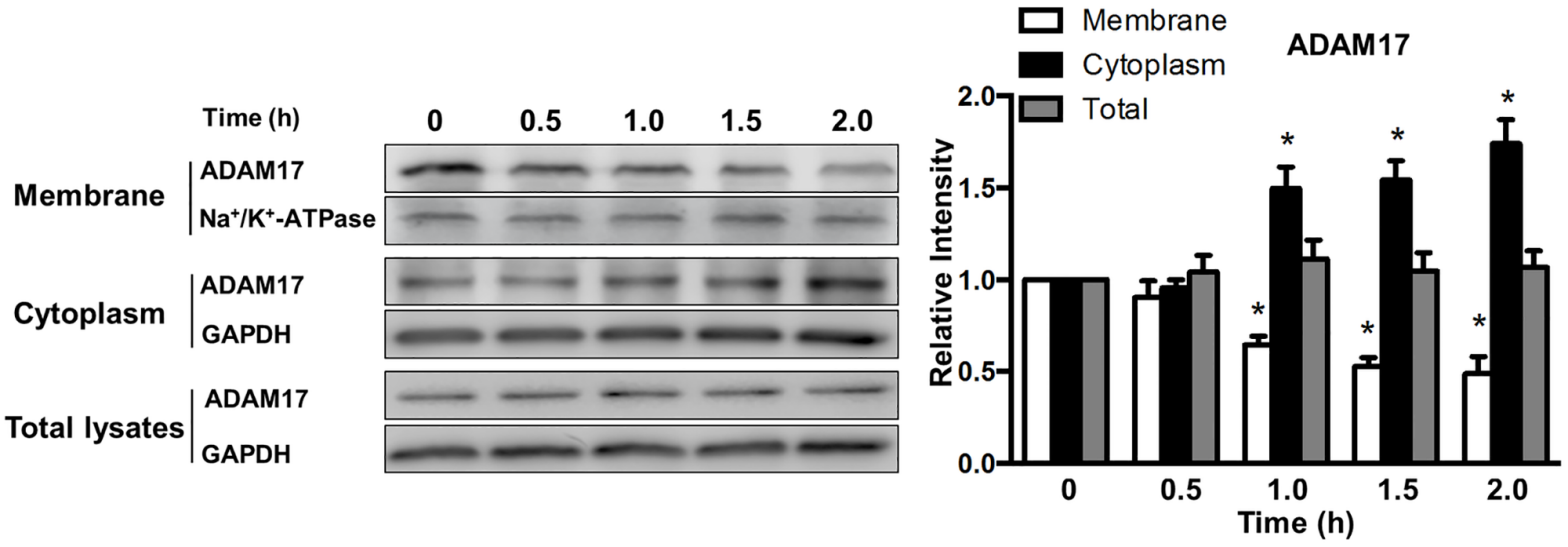
Effect of isoflurane on the total, surface and cytosolic expression of ADAM17 protein. BMDMs were treated with 1.0 MAC isoflurane for indicated time periods at 37°C. ADAM17 protein expression was analyzed by Western blot. Left, representative blots showing protein expression; right, quantification of ADAM17 protein expression by densitometry. Data are mean±SEM of 3 independent experiments. *P < 0.05 vs. corresponding control groups (no isoflurane).

The expression and function of cell surface receptors is tightly regulated by a proteolytic mechanism called ectodomain shedding through which the extracellular domain of receptors is released from the cell surface as soluble proteins [31]. A disintegrin and metalloprotease-17 (ADAM17), also termed TNF-α-converting enzyme (TACE), plays a broad role in ectodomain shedding of a variety of cell surface proteins including Mer [32]. We thus determined whether isoflurane regulates the transport of ADAM17 from the cytosol to cell surface by analyzing ADAM17 distribution following isoflurane treatment. Compared to control groups, ADAM17 expression exhibited a time-dependent decrease on the cell surface and a concomitant increase in its cytoplasmic fraction in response to isoflurane treatment. Isoflurane had no effect on total expression of ADAM17 (Fig 3). Our results demonstrate that isoflurane impedes the transport of ADAM17 from the cytosolic compartment to the cell surface.

### AMPK activation mediates macrophage efferocytosis following isoflurane treatment through ADAM17/Mer signaling

**Fig 4:**
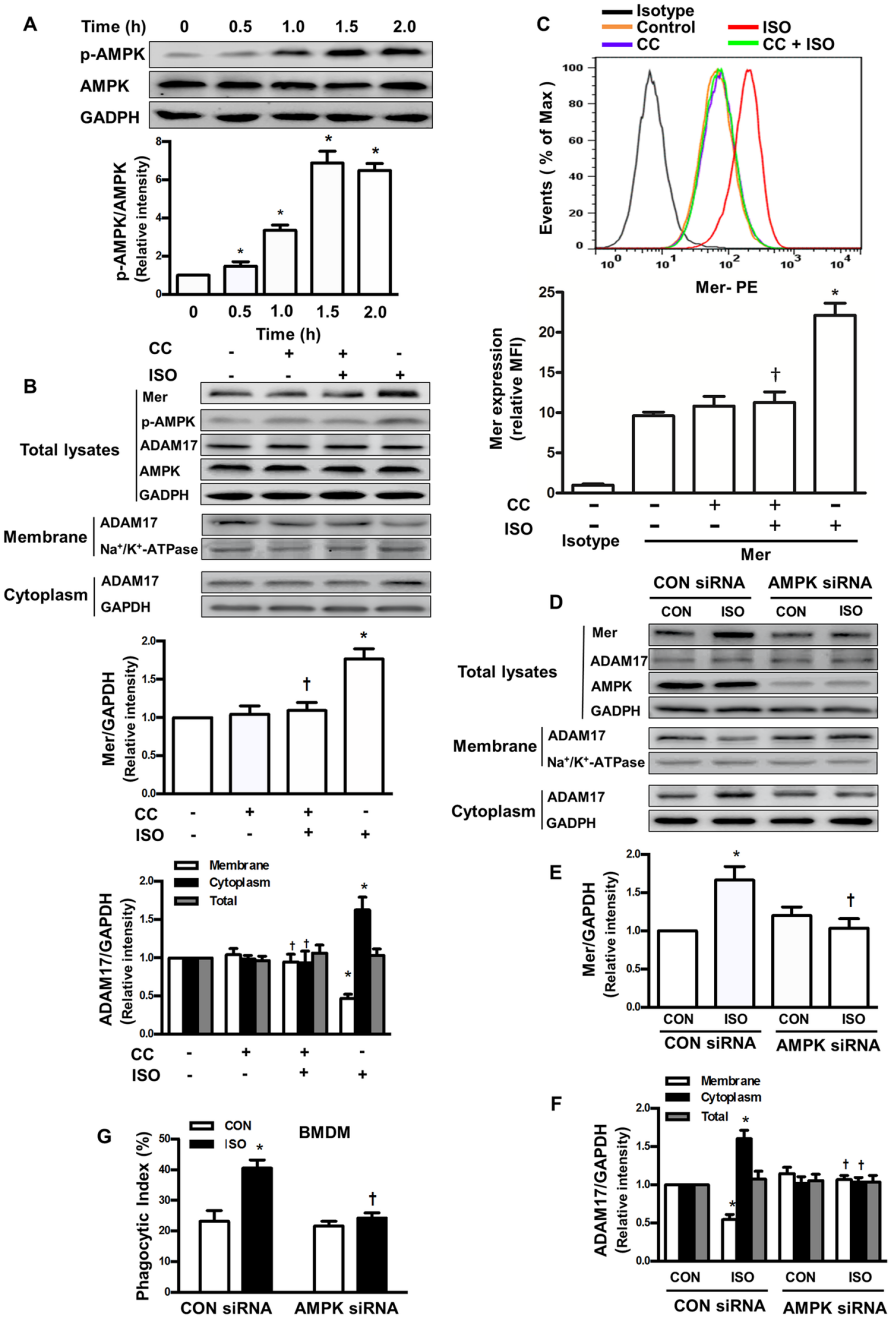
AMPK activation mediates macrophage efferocytosis following isoflurane treatment through ADAM17/Mer signaling in BMDMs. Murine BMDMs were treated with Compound C (CC, 40 μM) or transfected with an AMPK or control (CON) siRNA. Protein expression was analyzed by Western blot. (**A**) Effect of isoflurane (ISO) on AMPK and phospho-AMPK protein expression. Upper, representative blots showing protein expression; bottom, quantification of phospho-Mer protein expression by densitometry. (**B**) Effects of Compound C on protein expression of Mer, phospho-AMPK, total AMPK as well as the surface and cytosolic protein expression of ADAM17 following isoflurane treatment. Upper, representative blots showing protein expression; middle, quantification of Mer protein expression by densitometry; bottom, quantification of ADAM17 protein expression by densitometry. (**C**) Effects of Compound C (CC) on cell surface expression of Mer following isoflurane treatment. Top, surface expression of Mer was analyzed by flow cytometry. Top, representative flow cytometry histograms; bottom, quantitative data showing changes in relative MFI of Mer. (**D-F**) Effects of AMPK depletion on cell surface and cytosolic expression of ADAM17. D, representative blots showing protein expression; E, quantification of Mer protein expression by densitometry; F, quantification of ADAM17 protein expression by densitometry. (**G**) Effects of AMPK depletion on phagocytosis of apoptotic PMNs. n = 4–6 per group. *P < 0.05 vs. control groups (no isoflurane treatment, A, B and C) or control siRNA groups (no isoflurane treatment, E, F and G); †*P* < 0.05 vs. isoflurane alone groups (B and C) or corresponding isoflurane alone plus CON siRNA groups (E, F and G).

AMPK has been reported to regulate the cell surface proteome through modulation of the endomembrane traffic of proteins [33]. We therefore examined whether AMPK activity contributes to increased efferocytosis following isoflurane treatment through modulation of ADAM17-mediated cell surface Mer expression. As shown in Fig 4A, 1.0 MAC isoflurane induced AMPK phosphorylation (activation) in BMDMs in a time-dependent manner in comparison with vehicle, but had no effect on total AMPK expression. Complete suppression of AMPK with a selective inhibitor Compound C (6-[4-(2-Piperidin-1-ylethoxy) phenyl]-3-pyridin-4-ylpyrazolo [1,5-a]pyrimidine, 40 μM) [34] abolished isoflurane-induced increase in total and surface expression of Mer protein (Fig 4B). Analysis of cell fractionation indicated that compound C nearly completely reversed isoflurane-induced decrease in the surface expression of ADAM17 and increase in the cytosolic expression of ADAM17 (Fig 4B). Results from flow cytometry further showed that Compound C completely blocked isoflurane-induced increase in cell surface expression of Mer (Fig 4C). Compound C had no effect on the total expression of Mer and ADAM17 protein (Fig 4B) as well as Mer surface expression (Fig 4C) in the absence of isoflurane. Similarly, depletion of AMPK by a specific siRNA completely blocked total Mer expression caused by isoflurane (Fig 4D). AMPK knockdown also increased the surface expression of ADAM17, but decreased the cytosolic expression of ADAM17 following isoflurane treatment (Fig 4D–4F). Finally, AMPK knockdown completely abolished isoflurane-induced macrophage efferocytosis (Fig 4G). Thus, these findings reveal a new role for AMPK activation following isoflurane treatment in mediating macrophage efferocytosis through ADAM17/Mer signaling.

### Isoflurane enhances clearance of apoptotic PMNs and improves LPS-induced acute lung injury

**Fig 5:**
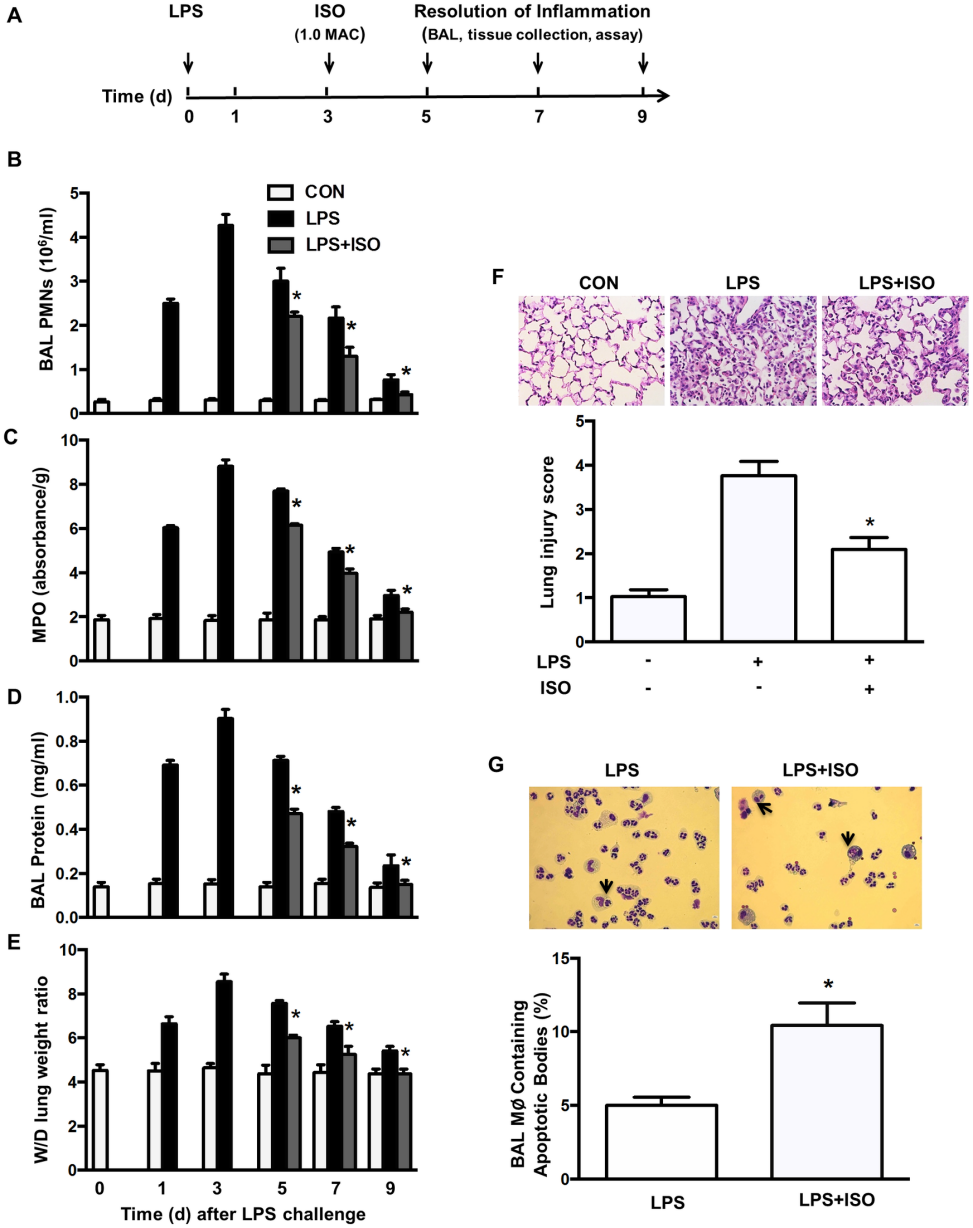
Treatment of isoflurane enhances macrophage efferocytosis and facilitates resolution of lung inflammation and injury following LPS challenge. (**A**) Experimental protocols of induction and time course of resolution of lung inflammation post-LPS or vehicle (CON) challenge in wild type mice. Mice were exposed to 1.0 MAC isoflurane (ISO) or air in an airtight box for 1 h. (**B**) PMN counts in the BAL fluid. (**C**) PMN sequestration in lungs as evaluated by MPO activity. (**D**) Pulmonary vascular protein permeability as determined by protein concentration of BAL fluid. (**E**) Pulmonary edema formation measured by wet-to-dry (W/D) lung weight ratio. (**F**) Histology of lung lesions. Upper, lung tissue stained by hematoxylin and eosin staining (magnification, 40×); bottom, histopathological mean lung injury scores from low-power (×20) sections. (**G**) Efferocytosis by alveolar macrophages. Upper, representative photomicrographs depicting cytospin preparations of BAL cells 2 d after intratracheal injection of BMDMs. Arrows indicate macrophages containing apoptotic bodies. Original magnification, 40×. Bottom, quantification of macrophages (Mϕ) containing apoptotic bodies in BAL fluid. The count of total macrophages in BAL fluid: CON group, (483.3±17.64) ×10^4^; LPS group, (0.943±0.052)×10^4^; and LPS+ISO group, (0.907±0.020)×10^4^. n = 6 animals/group/time point. *P < 0.05 vs. corresponding LPS groups. n = 6 animals/group/time point. *P < 0.05 vs. corresponding LPS groups.

**Fig 6:**
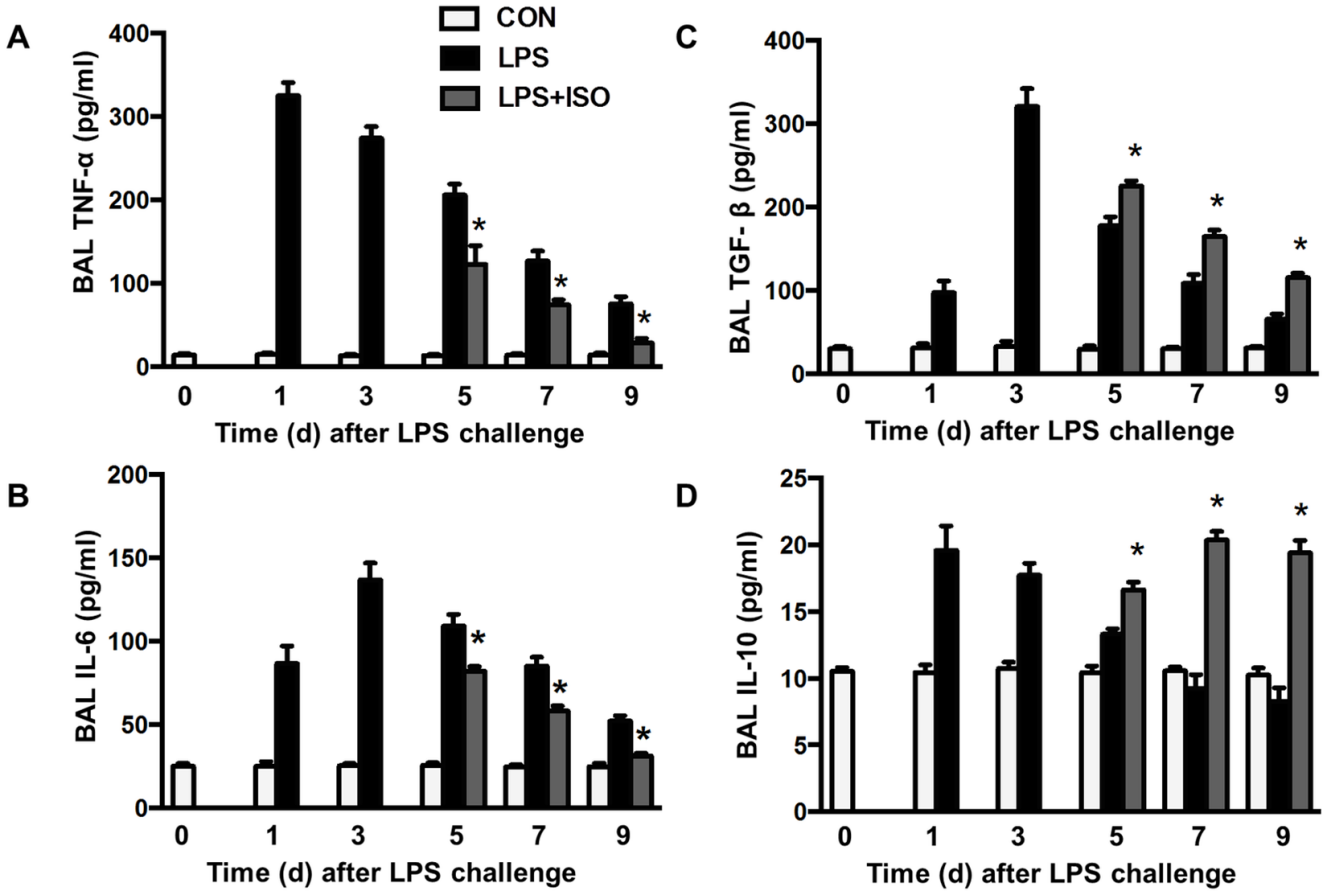
Effects of isoflurane on the release of cytokines in the lung following LPS challenge. Experimental protocols of induction and time course of resolution of lung inflammation post-LPS or vehicle (CON) challenge in wild type mice were shown as Fig 5A. Mice were exposed to 1.0 MAC isoflurane (ISO) in an airtight box for 1 h. (**A-D**) The levels of TNF-α (A), IL-6 (B), TGF-β1 (C) and IL-10 (D) in BAL fluid measured by ELISA. n = 6 animals/group/time point. *P < 0.05 vs. corresponding LPS groups.

Clearance of apoptotic PMNs favors resolution of inflammation by down-regulating the inflammatory phenotype in activated macrophages [2]. Because isoflurane increased phagocytosis of apoptotic PMNs (efferocytosis) *in vitro*, we tested the possibility that isoflurane speeds up resolution of lung inflammation through positive regulation of efferocytosis in an *in vivo* model of LPS-induced lung inflammation (Fig 5A). Following LPS challenge, PMN counts in BAL fluid (Fig 5B), lung MPO levels (Fig 5C), protein levels in BAL fluid (Fig 5D), and edema formation (Fig 5E) in control mice were increased, reached peak levels at d 3 and then gradually reduced thereafter. Treatment of mice with isoflurane at d 3 following LPS challenge accelerated the resolution of neutrophil infiltration (Fig 5B and 5C), exuded protein (Fig 5D), and lung edema (Fig 5E) at d 5, 7 and 9. Lung tissues showed severe histological damage characterized by alveolar congestion, exudates, and infiltration of inflammatory cells at d 5 following LPS challenge (Fig 5F). These histopathological changes in lung tissues were significantly improved by treatment of isoflurane, as seen in the lower lung injury score (Fig 5F). Correspondingly, the number of macrophages containing apoptotic bodies or cells in BAL fluid was much higher in isoflurane-treated lungs than in vehicle-treated lungs at d 2 after injection of BMDMs (d 5 following LPS challenge) (Fig 5G). The levels of proinflammatory cytokines TNF-α (Fig 6A) and IL-6 (Fig 6B) in the BAL fluid were elevated and reached peak levels at d 1 (TNF-α) or 3 (IL-6) following LPS challenge and then gradually decreased in control mice. Isoflurane-treated mice showed lower levels of TNF-α (Fig 6A) and IL-6 (Fig 6B) compared to vehicle-treated mice at d 5, 7 and 9 following LPS challenge. In contrast, isoflurane-treated mice had a remarkably increased levels of anti-inflammatory cytokines TGF-β1 (Fig 6C) and IL-10 (Fig 6D) compared with those treated with vehicle at d 5, 7 and 9 following LPS challenge. These data clearly show that isoflurane treatment accelerates resolution of LPS-induced lung inflammatory injury and increases efferocytosis *in vivo*.

**Fig 7:**
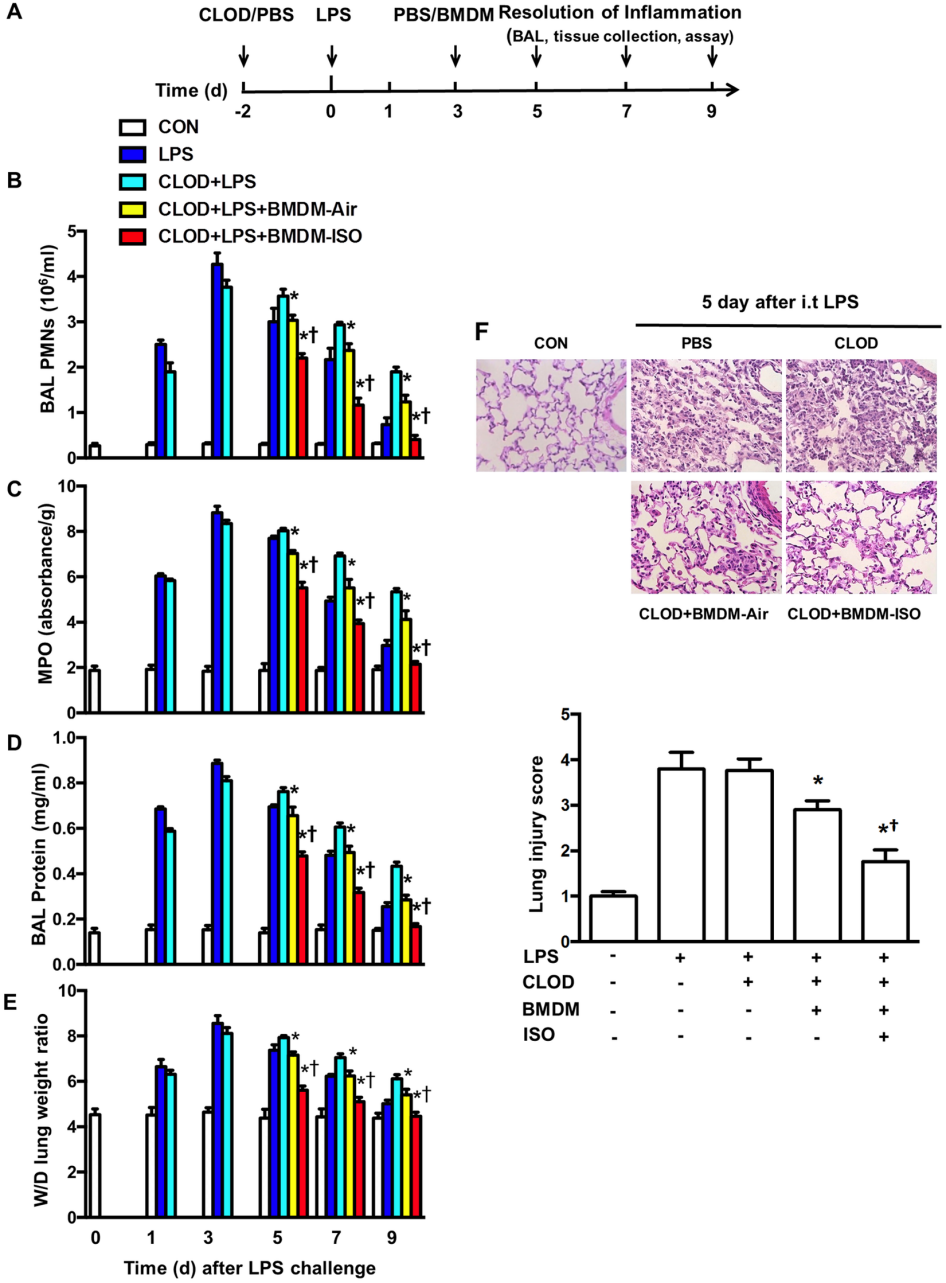
Transplantation of isoflurane-pretreated BMDMs facilitates resolution of lung inflammation and injury following LPS challenge. (**A**) Experimental protocols of induction and time course of resolution of lung inflammation post-LPS or vehicle (CON) challenge in wild type mice. Following depletion of alveolar macrophages (AMs) with clodronate liposomes (CLOD) in mice, acute lung injury was induced by intratracheal instillation of LPS. BMDMs isolated from donor mice were cultured and treated with 1.0 MAC isoflurane (ISO) or air for 1 h. BMDMs pretreated with air or isoflurane were intratracheally injected into AM-depleted mice. Resolution of lung inflammatory injury was determined at day 1, 3, 5, 7 and 9 post LPS challenge as described in *Materials and Methods*. n = 6 animals/group/time point. (**B**) PMN counts in the BAL fluid. (**C**) PMN sequestration in lungs as measured by MPO activity. (**D**) Pulmonary vascular protein permeability as estimated by protein concentration of BAL fluid. (**E**) Pulmonary edema formation measured by wet-to-dry (W/D) lung weight ratio. (**F**) Histology of lung lesions. Upper, lung tissue by hematoxylin and eosin staining (magnification, 40×); bottom, histopathological mean lung injury scores from low-power (magnification, 20×) sections. n = 6 animals/group/time point. *P < 0.05 vs. corresponding LPS groups; †*P* < 0.05 vs. CLOD+LPS (day 3) groups.

**Fig 8:**
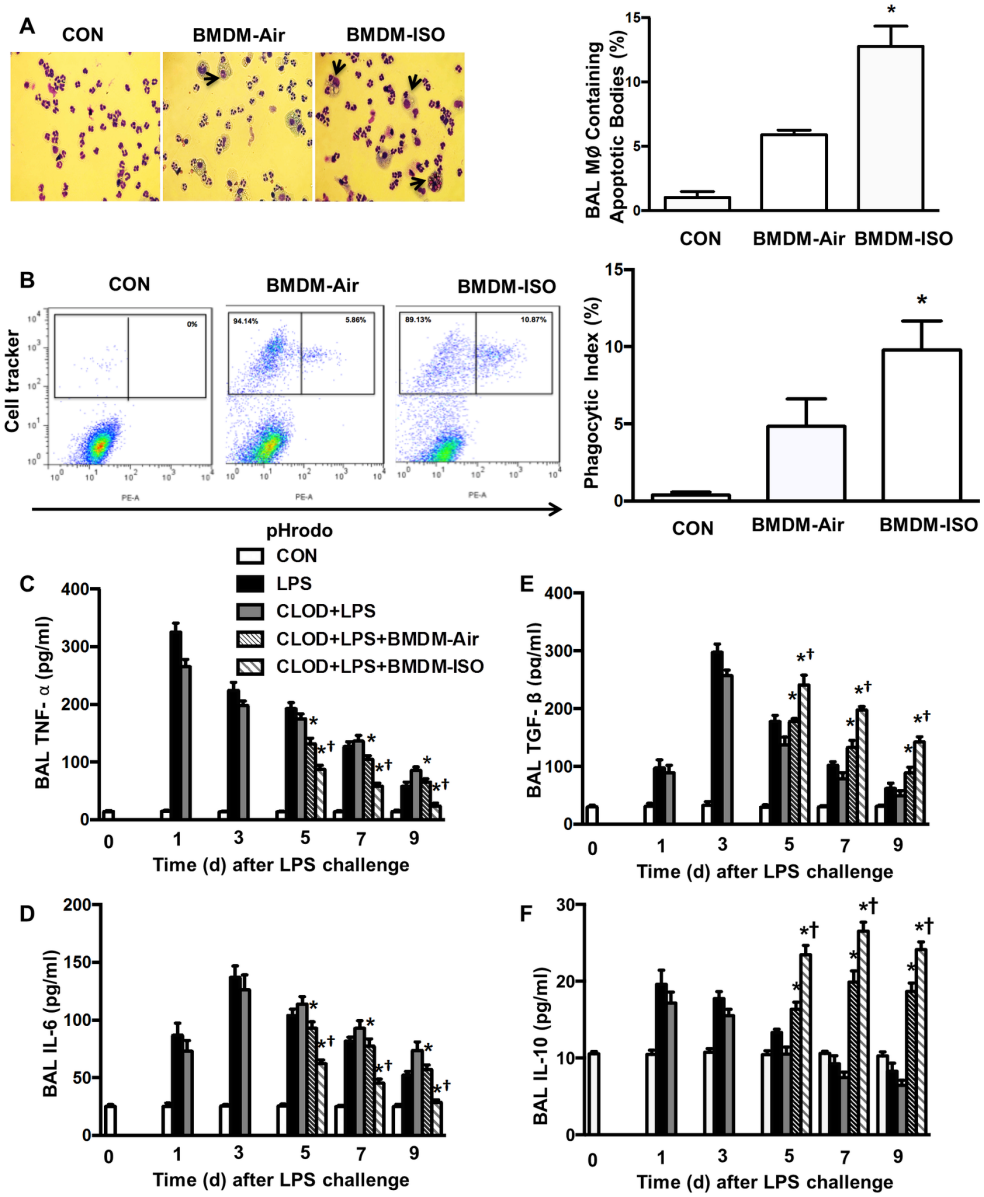
Effects of transplantation of isoflurane-pretreated BMDMs on efferocytosis and the release of cytokines in the lung following LPS challenge. Experimental protocols of induction and time course of resolution of lung inflammation post-LPS challenge in wild type mice were shown as Fig 7A. Alveolar macrophage-depleted mice were intratracheally administered with vehicle (CON), or BMDMs pretreated with air or 1.0 MAC isoflurane (ISO). (**A**) Efferocytosis by alveolar macrophages. Left: representative photomicrographs of cytospin preparations of BAL cells 2 d after injection of BMDMs. Arrows indicate macrophages containing apoptotic bodies. Original magnification, 40×. Right, quantification of macrophages (Mϕ) containing apoptotic bodies in BAL fluid. The count of total macrophages in BAL fluid: CON group, (0.0364±0.0015) × 10^4^; BMDM-Air group, (0.530±0.021) × 10^4^; and BMDM-ISO group, (0.547±0.038) × 10^4^. (**B**) Effects of isoflurane pretreatment on phagocytosis of apoptotic PMNs *in vivo* following LPS challenge. 1.0 × 10^7^ pHrodo^™^ Red (SE)-labeled apoptotic PMNs were intratracheally instilled 2 d following CellTracker^™^ Green- labeled BMDMs transplantation (2.0 × 10^6^). At 4 h after instillation of apoptotic PMNs, BAL was performed, washed, and analyzed by flow cytometry. Left, representative flow cytometric dot plots demonstrating changes in the proportion of macrophages engulfing pHrodo-stained apoptotic PMNs are shown; right, phagocytic index was calculated by average percent of macrophages containing apoptotic PMNs. n = 6 animals/group/time point. *P < 0.05 vs. corresponding LPS groups; †*P* < 0.05 vs. CLOD+LPS (day 3) groups. (**C-F**) The levels of TNF-α (C), IL-6 (D), TGF-β1 (E) and IL-10 (F) in BAL fluid measured by ELISA. n = 6 animals /group/time point. *P < 0.05 vs. corresponding LPS groups. †*P* < 0.05 vs. CLOD+LPS (day 3) groups.

To further elucidate whether increased alveolar macrophage efferocytosis plays a crucial role in promoting resolution of LPS-induced lung inflammation by isoflurane, a recently developed *in vivo* model [26] was utilized. By transplanting BMDMs treated with vehicle or isoflurane into alveolar macrophage-depleted mice, we specifically examined the effect of increased macrophage efferocytosis induced by isoflurane on resolution of LPS-induced lung inflammatory injury. Briefly, mice were first depleted of alveolar macrophages and then challenged with LPS, followed by intratracheal delivery of BMDMs pretreated with vehicle or isoflurane (Fig 7A). As shown in our previous study [26], the spatial distribution of transplanted macrophages following intratracheal instillation in alveolar macrophage-depleted lungs receiving BMDMs treated with vehicle or isoflurane is uniform and commensurate. Consistent with our prior findings [26], PMN counts in BAL fluid (Fig 7B), lung MPO levels (Fig 7C), protein levels in BAL fluid (Fig 7D), and edema formation (Fig 7E) following LPS challenge in control mice (without alveolar macrophage depletion) and alveolar macrophages-depleted mice were increased, reached peak levels at d 3 and then gradually declined. Mice depleted of alveolar macrophages exhibited the postponed resolution of neutrophil infiltration (Fig 7B and 7C), exuded protein (Fig 7D), and lung edema (Fig 7E) at d 7 and 9 following LPS challenge whereas these delayed responses were reversed by administration of vehicle-treated BMDMs (Fig 7B–7E), indicating the important role of macrophages in resolution of lung inflammation. Importantly, alveolar macrophage-depleted mice receiving isoflurane-treated BMDMs showed further decreased PMN counts in BAL fluid (Fig 7B), lung MPO (Fig 7C), protein level in BAL fluid (Fig 7D), and edema formation (Fig 7E) compared with mice receiving vehicle-treated BMDMs at d 5, 7 and 9 following LPS challenge. Lung tissues from control mice (without alveolar macrophage depletion) and alveolar macrophages-depleted mice had severe histological changes, including alveolar congestion, exudates, and infiltration of inflammatory cells at d 5 following LPS challenge, in comparison with untreated mice (Fig 7F). These histopathological alterations in lung tissues were ameliorated following administration of vehicle-treated BMDMs and further dramatically improved by administration of isoflurane-treated BMDMs, as seen in the significantly reduced lung injury score (Fig 7F). Consistently, at d 2 after injection of BMDMs (d 5 following LPS challenge), the number of macrophages containing apoptotic bodies or cells in BAL fluid was much higher in isoflurane-treated lungs than in vehicle-treated lungs (Fig 8A). To further demonstrate the role of isoflurane in the clearance of apoptotic PMNs by macrophages *in vivo*, apoptotic mouse pHrodo^™^ Red (SE)-labeled PMNs were intratracheally injected into mice at d 2 following delivery of BMDMs (d 5 following LPS challenge) and BAL fluid harvested 3 h later for determination of macrophage phagocytosis of PMNs. As shown in Fig 8B, the number of macrophages containing apoptotic PMNs in BAL was greater in mice receiving isoflurane-treated BMDMs than in those receiving vehicle-treated BMDMs. The levels of proinflammatory cytokines TNF-α (Fig 8C) and IL-6 (Fig 8D) in the BAL fluid were elevated and reached peak levels at d 1 (TNF-α) or 3 (IL-6) following LPS challenge and then gradually decreased thereafter in control mice (without alveolar macrophage depletion) and alveolar macrophages-depleted mice. At d 5, 7, and 9 following LPS challenge, alveolar macrophage—depleted mice receiving vehicle-treated BMDMs showed lower levels of TNF-α (Fig 8C) and IL-6 (Fig 8D), which further decreased in alveolar macrophage—depleted mice receiving isoflurane-treated BMDMs (Fig 8C and 8D). Alveolar macrophage—depleted mice receiving isoflurane-treated BMDMs had a remarkably increased level of anti-inflammatory cytokines TGF-β1 (Fig 8E) and IL-10 (Fig 8F) compared with those receiving vehicle-treated BMDMs at days 5, 7, and 9 following LPS challenge. Collectively, these data strongly suggest that isoflurane promotes resolution of lung inflammation and injury during sepsis by enhancement of macrophage efferocytosis.

## Discussion

The findings in this study highlight the therapeutic potential of the volatile anesthetic isoflurane as a novel candidate for promoting resolution of lung inflammation during the perioperative period. We have shown that isoflurane facilitated the clearance of apoptotic PMNs both *in vitro* and *in vivo* and markedly stimulated the release of anti-inflammatory cytokines and accelerated recovery of lung inflammatory injury. Furthermore, we provide a comprehensive mechanistic framework for how isoflurane up-regulates the ability of macrophages to phagocytose apoptotic PMNs, including a crucial role for AMPK activation, decreased ADAM17 trafficking to the cell membrane and increased Mer surface expression.

We for the first time showed that isoflurane at the concentration of 1.0 MAC increased phagocytosis of apoptotic PMNs in murine alveolar macrophages and BMDMs. A previous study reported that the phagocytosis of unopsonized and opsonized particles (1 μm diameter) by human alveolar macrophages decreased during anesthesia with isoflurane [30]. These findings suggest that the impact of isoflurane on the function of phagocytosis by macrophages depends mainly on the feature of materials to be engulfed. In support of this concept, LPS and TNF-α have been shown to decrease phagocytosis of apoptotic PMNs but not IgG-opsonized erythrocytes [35]. Phagocytic receptor expression on the surface of macrophages is a key determinant in the internalization process. Engagement of efferocytic receptors initiates signaling events responsible for phagocytosis of apoptotic cells, whereas Fcγ-receptor-mediated phagocytosis relies on the sequential interaction of IgG-coated particles with receptors [36]. It is thus likely that isoflurane deferentially modulates the phagocytic function of macrophages via its effect on different receptors.

Our results identified the crucial role of a signaling pathway involving Mer receptors in isoflurane-induced enhancement of macrophage efferocytosis. Mer receptor binds externalized PS on apoptotic cells through bridging molecules Gas6 and mediates a key process in efferocytosis and inflammation resolution [37]. Either Mer depletion or Mer cleavage decreased macrophage efferocytosis of PMNs and delayed resolution of Zymosan-induced peritonitis [38]. The ectodomain of Mer at proline 485 in murine macrophages can be cleaved by the metalloproteinase ADAM17. The cleavage product, soluble Mer, competitively inhibits the interaction of intact Mer with its ligands Gas6 [39, 40]. In the present study, we found that isoflurane increased the total and surface expression of Mer in BMDMs without a change in cytosolic level of Mer, raising the possibility that isoflurane suppresses Mer cleavage. Consistently, isoflurane significantly reduced the surface expression of ADAM17 but increased cytosolic level of ADAM17, suggesting that ADAM17 accumulated in the cytosol and was unable to transport to the cell membrane. Our results strongly indicate that isoflurane at clinical concentrations promotes macrophage efferocytosis via enhanced surface expression of Mer by blockade of ADAM17 trafficking to cell membrane.

While the activation and trafficking of ADAM17 has recently been reported [41, 42], little is known about how isoflurane prevents ADAM17 trafficking to the cell membrane from the cytosol. Our findings clearly show the critical role for AMPK activation in the regulation of ADAM17 trafficking following isoflurane treatment. We first found that isoflurane induced AMPK activation in BMDMs, consistent with the previous finding from cardiomyocytes [43]. Importantly, either genetic depletion of AMPK or a specific pharmacological inhibitor completely abolished a decrease in ADAM17 surface expression and an increase in cytosolic level of ADAM17 in response to isoflurane treatment. These effects coupled with the changes in Mer surface expression and phagocytosis of apoptotic PMNs. Taken together, our results indicate that isoflurane induces AMPK activation which blocks ADAM17 trafficking to the cell membrane, preventing Mer cleavage. Isoflurane-induced upregulation of Mer surface expression in turn leads to an increase in efferocytosis by macrophages. Another interesting question arising from these findings is about how AMPK activation regulates ADAM17 trafficking in macrophages. A proteolytically inactive member of the rhomboid family, iRhom2 (RHBDF2), was shown to interact with ADAM17 and promotes its exit from the endoplasmic reticulum. iRhom2 is critical for ADAM17 maturation and trafficking to the cell surface in hematopoietic cells [41, 42]. It is therefore speculated that isoflurane-induced AMPK activation may disrupt the interaction between iRhoma2 and ADAM17, blocking ADAM17 trafficking to the cell membrane. Although AMPK activation has been shown to enhance macrophage efferocytosis through modulation of cytoskeletal reorganization [44], our findings clearly demonstrate an important role for AMPK activation in the regulation of ADAM17-mediated surface expression of Mer receptor and subsequent efferocytosis following isoflurane treatment.

Two preclinical mouse models were intentionally used to examine the potential therapeutic effects of isoflurane at a clinically relevant concentration on the resolution of LPS-induced lung inflammation and injury. In one mouse model of LPS-induced lung inflammatory injury, mice were inhaled with 1.0 MAC isoflurane for 1 h on day 3 after LPS challenge. The purpose of this experimental design was to test whether conventional inhalation of isoflurane accelerates resolution of lung inflammation. We observed that administration of isoflurane caused a dramatic decrease in PMN infiltration into the lung, protein-rich edema formation, lung tissue injury. These findings clearly demonstrate the ability of isoflurane to accelerate resolution of lung inflammation and injury while it was used after LPS challenge. To further expound whether the beneficial effect of isoflurane is mainly due to its action on macrophage efferocytosis, we chose the alveolar macrophage-depleted mouse model of LPS-induced lung injury to determine the effect of pulmonary transplantation of isoflurane-pretreated BMDMs on the resolution of lung inflammation [26]. As expected, intratracheal administration of isoflurane-pretreated BMDMs significantly promoted resolution of lung inflammation and facilitated repair of lung injury, which intimately tied to increased phagocytosis of apoptotic PMNs and bodies by alveolar macrophages. Efficient efferocytosis represents a critical step in tissue remodeling, modulation of immune responses, and resolution of inflammation. The impairment of efferocytosis is directly correlated with increased morbidity and mortality after lung inflammatory injury [45–48]. Decreased efferocytosis has also been closely associated with both acute and chronic pulmonary inflammatory diseases [2]. Taken together, our *in vivo* and *in vitro* findings strongly support the notion that isoflurane accelerates resolution of LPS-induced lung inflammation and injury through modulation of efferocytosis. Thus, utilization of volatile anesthetic isoflurane during surgery may improve outcomes in patients with sepsis.

Phagocytosis of apoptotic PMNs can stimulate macrophages to release cytokines that inhibit the inflammatory response and favor resolution of inflammation (1–4). In this study, enhanced efferocytosis by macrophages upon isoflurane exposure is coupled with higher levels of anti-inflammatory cytokines TGF-β1 and IL-10 and lower levels of proinflammatory cytokines TNF-α and IL-6. Macrophage-derived TGF-β plays a crucial role in resolution of lung inflammation due to its potent regulatory and anti-inflammatory activities, and its role in epithelial restitution and fibrosis [8]. IL-10 not only directly blocks the production of proinflammatory cytokines TNF-α and IL-6 by macrophages, but also induces the conversion of inflammatory M1 macrophages into the anti-inflammatory anti-inflammatory M2 macrophages and further enhances efferocytosis [49]. Thus, increased efferocytosis by alveolar macrophages following isoflurane treatment may trigger an anti-inflammatory response and resolution of lung inflammation through the induction of TGF-β1 and IL-10.

The use of clodronate to specifically deplete alveolar macrophages (and not dendritic cells or PMNs) [50], adds specificity to the importance of macrophages in the resolution of lung inflammation. Our results indicated that depletion of alveolar macrophages significantly delayed resolution of lung inflammation, consistent with the previous finding that depletion of lung macrophages during the priming response completely abrogated the positive effect of immunological priming on resolution of lung inflammation [51]. In our model, lung injury peaked on day 3 after LPS challenge before the resolution of lung inflammation proceeded. At that time administration of untreated BMDMs was able to promote recovery of lung inflammatory injury, further suggesting the important role of macrophages in tissue repair [26]. Interestingly, pretreatment of macrophages with isoflurane enhanced the ability of macrophages to remove apoptotic PMNs and accelerate resolution of lung inflammation and injury, suggesting the specific impact of isoflurane on macrophage-mediated resolution of inflammatory processes.

**Fig 9:**
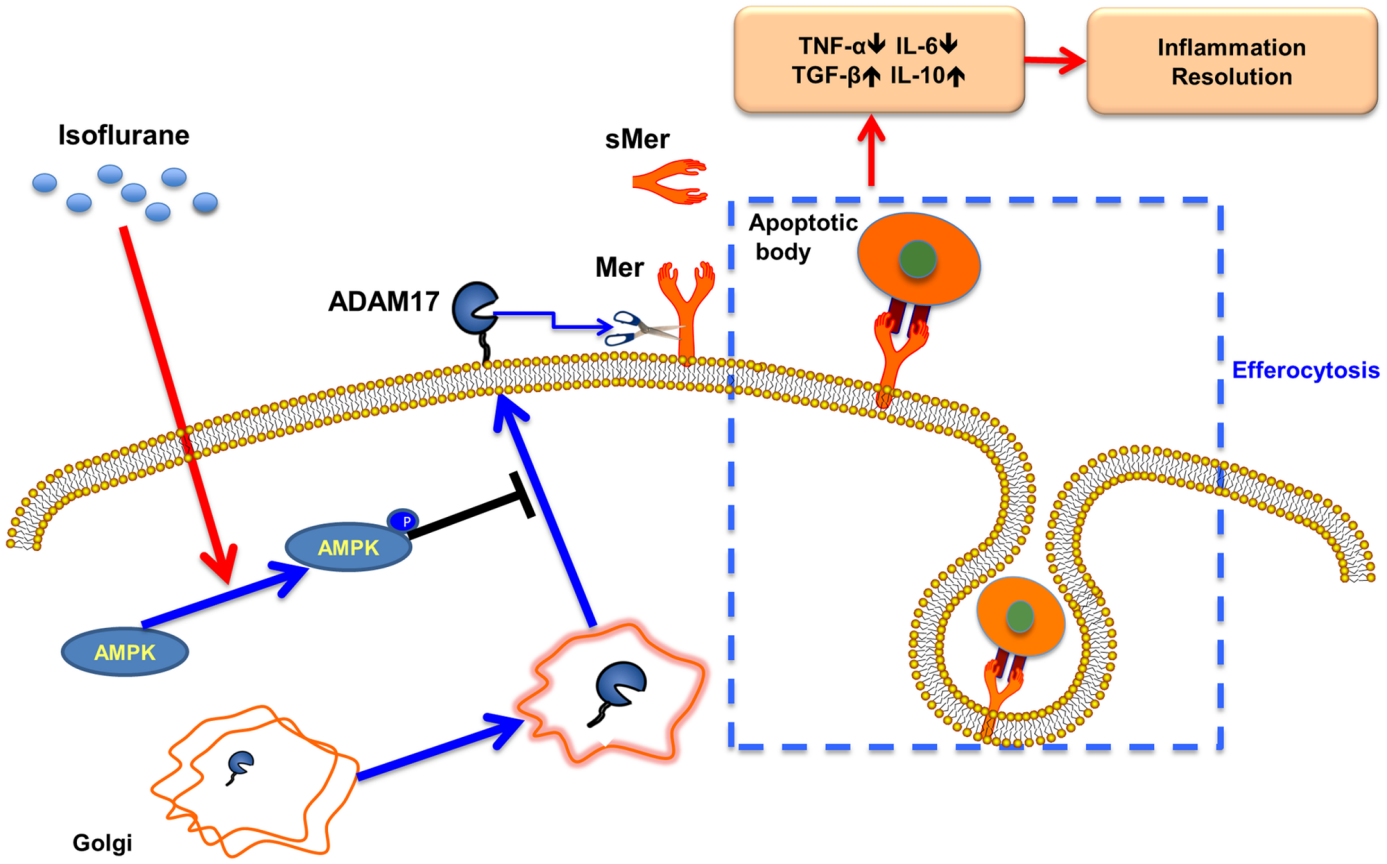
A model showing the effect of isoflurane on macrophage efferocytosis. sMer = soluble Mer.

In conclusion, we demonstrate what we believe to be an innovative therapeutic effect of the volatile anesthetic isoflurane for the resolution of sepsis-induced lung inflammation and injury. Isoflurane induces AMPK activation in macrophages, which results in blockade of ADAM17 trafficking to the cell membrane where Mer cleavage is abolished. Increased surface expression of Mer receptors by isoflurane enhances macrophage efferocytosis, which subsequently accelerates resolution of lung inflammation and injury during sepsis (Fig 9). The ability of isoflurane to upregulate macrophage efferocytosis represents a novel mechanism of action for this volatile anesthetic. Clinical use of isoflurane in patients with sepsis during operations may be helpful for the recovery from inflammation and tissue injury.
